# Supplementary material for: Cytometry-based single-cell analysis of intact epithelial signaling reveals MAPK activation divergent from TNF-α-induced apoptosis in vivo
Source: Mol Syst Biol. 2015 Oct 30;11(10):835. doi: 10.15252/msb.20156282 (PMC4631206; doi:10.15252/msb.20156282)
Supplement: Supplementary file 1 — Appendix [file msb0011-0835-sd1.pdf]

Cytometry-based single cell analysis of intact epithelial signaling reveals MAPK activation divergent from TNF-α-induced apoptosis in vivo

Alan J. Simmons, Amrita Banerjee, Eliot T. McKinley, Cherie' R. Scurrah, Charles A. Herring, Leslie S. Gewin, Ryota Masuzaki, Seth J. Karp, Jeffrey L. Franklin, Michael J. Gerdes, Jonathan M. Irish, Robert J. Coffey, Ken S. Lau

Appendix

1. Figure S1
2. Figure S2
3. Figure S3
4. Figure S4
5. Figure S5
6. Figure S6
7. Figure S7
8. Figure S8
9. Figure S9
10. Figure S10
11. Table S1

Appendix Figure S1

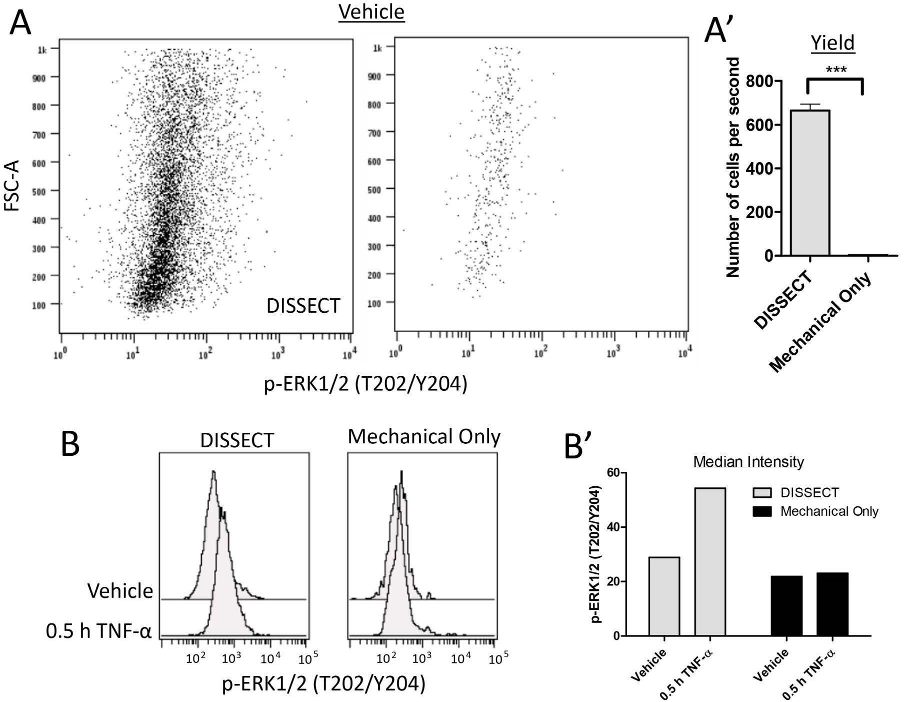

Appendix Figure S1: Conventional disaggregation using mechanical dissociation only without enzymes results in low yield and does not preserve signal transduction. (A) Single cells resulting from DISSECT or mechanical dissociation only detected by flow cytometry. Same amount of starting duodenal material was used. DISSECT sample was collected for 10 seconds while Mechanical Only sample was collected for 4 minutes by flow cytometry. (A') Yield of cells normalized to number of cells detected per second by flow cytometry. Error bars represent n=4 different animals. *** $P \leq 0.001$ (B) Stimulation of p-ERK, detected by our best working antibody, in the duodena of mice treated with vehicle or TNF-α for 0.5 h processed using DISSECT or mechanical dissociation only. (B') Quantification of median cell intensity from data generated from A. The stimulated p-ERK signal is preserved by DISSECT, but not by disaggregation by mechanical dissociation only.

Appendix Figure S2

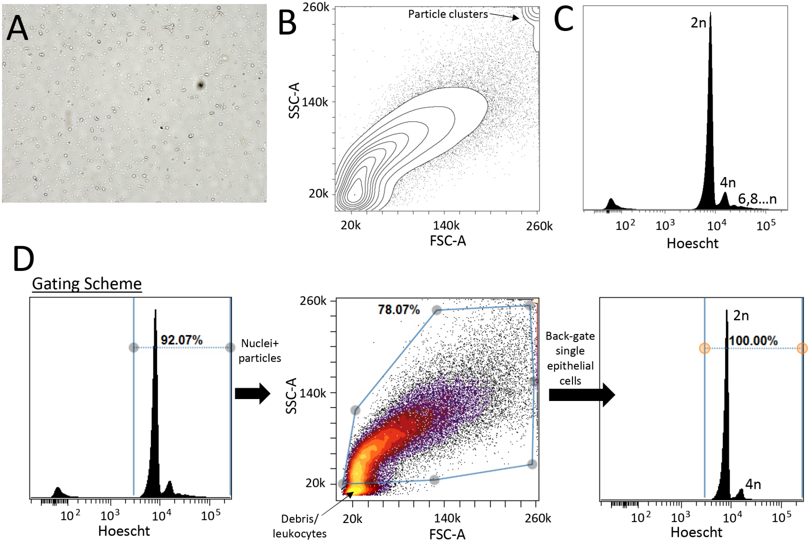

Appendix Figure S2: DISSECT generates single cell suspensions. (A) Light microscopy of a single cell suspension prepared by DISSECT. (B) Contour bi-plot for forward and side scatter of a DISSECT single cell prep. (C) DNA content indicated by Hoescht of a DISSECT single cell prep prior to gating. (D) Gating by DNA content and particle size to examine only single cells in the suspension, with most particles post-gating displaying 2n/4n DNA content.

Appendix Figure S3

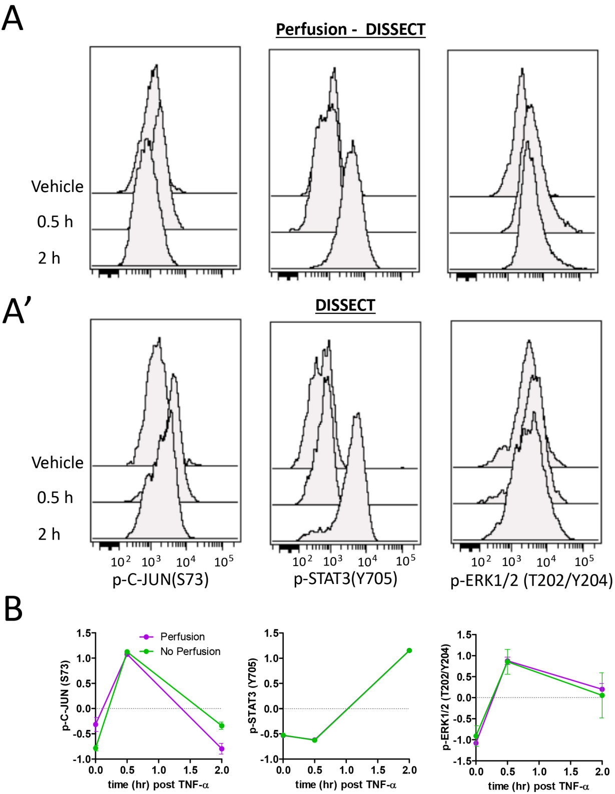

Appendix Figure S3: Perfusion with fixative does not improve signal preservation. (A) An example of the typical dynamics of p-C-JUN, p-STAT3, and p-ERK1/2 stimulation in TNF-α exposed duodenum tissues with (A) or without (A') an added perfusion step to most accurately maintain native signals. Samples were then prepared with DISSECT for flow cytometry. (B) Comparison of quantitative data derived from samples prepared with perfusion and without perfusion. See Supplementary Figure 3 for data normalization and scales. Error bars represent SEM from biological duplicates.

Appendix Figure S4

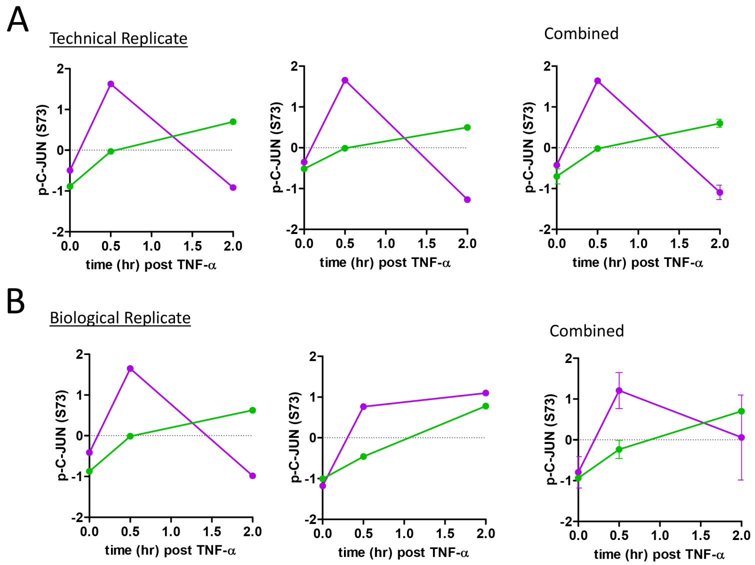

Appendix Figure S4: DISSECT performance on replicates. (A) Technical replicates. (B) Biological replicates.

Appendix Figure S5

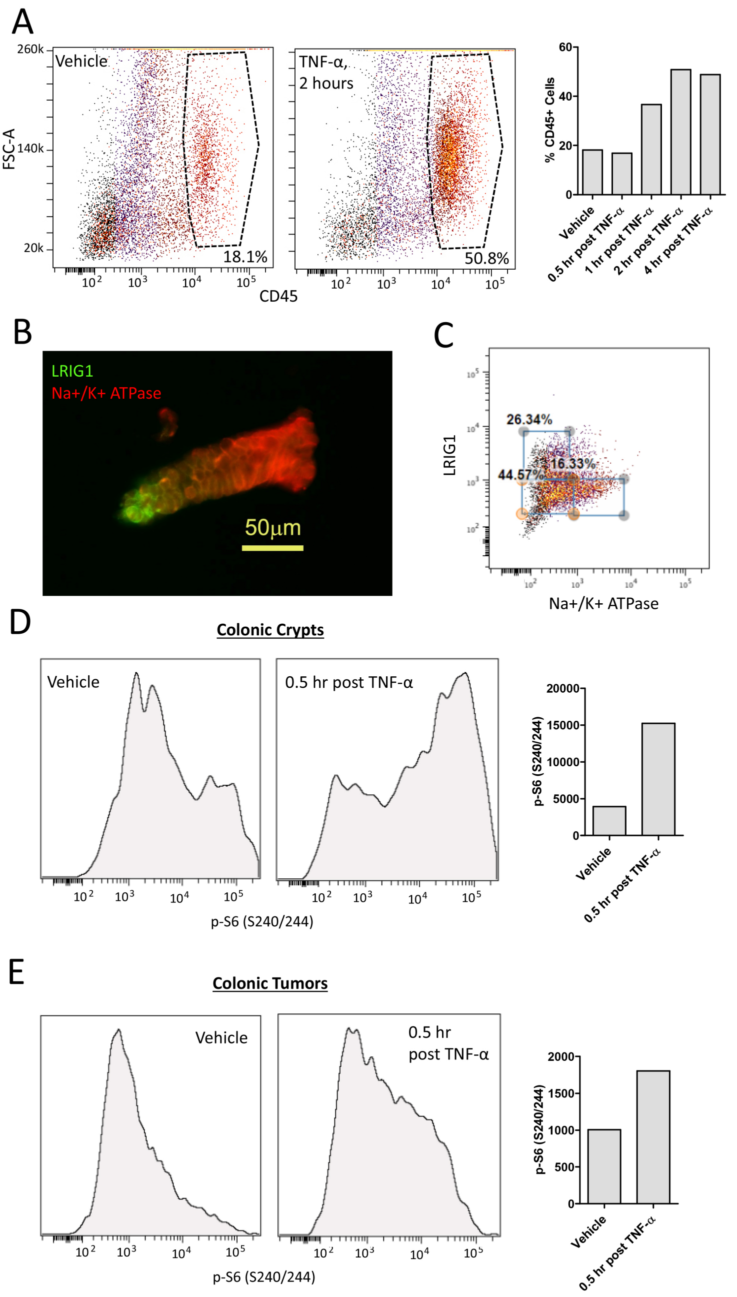

Appendix Figure S5: DISSECT maintains cell identity markers and its performance in other biological contexts. (A) DISSECT-flow cytometry quantification of CD45+ leukocytes in the intestine for a time course of TNF-α stimulation. TNF-α is a proinflammatory cytokine that has been shown to induce immune cell infiltration to the gut. (B) Stem cell surface marker LRIG1 and membrane Na+/K+ ATPase visualized by IF in colonic crypts. Note the anti-correlation in expression. (C) Quantification of LRIG1 and Na+/K+ ATPase-positive cells by DISSECT-flow cytometry, demonstrating the observed anti-correlation. (D-E) DISSECT-flow cytometry quantification of p-S6 stimulated by systemic TNF-α in (D) colonic crypts, and in a (E) Lrig1$^{CreERT2/+}$;Apc$^{fl/+}$ colonic tumor.

Appendix Figure S6

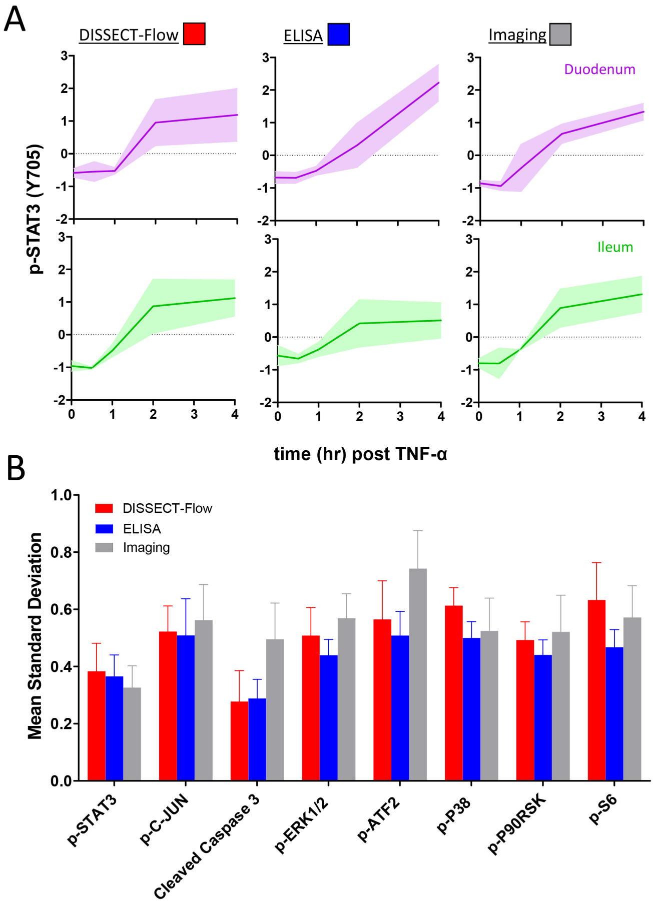

Appendix Figure S6: Quantitative data generated by DISSECT-flow cytometry contains the same level of noise as data from lysate-based approaches. (A) Standard deviation (SD - shading) of quantitative p-STAT3 data over a TNF-α time course between the duodenum and ileum obtained by DISSECT-Flow, ELISA and Imaging. n=3 animals per time point. (B) Comparison of the level of standard deviation for each signaling marker between the three quantification methods. The Mean Standard Deviation was calculated by averaging the standard deviation from the time points from TNF-α time courses generated from the duodenum and ileum (5 time points each). Hence, the error bars represent SEM of n=10 SD values.

Appendix Figure S7

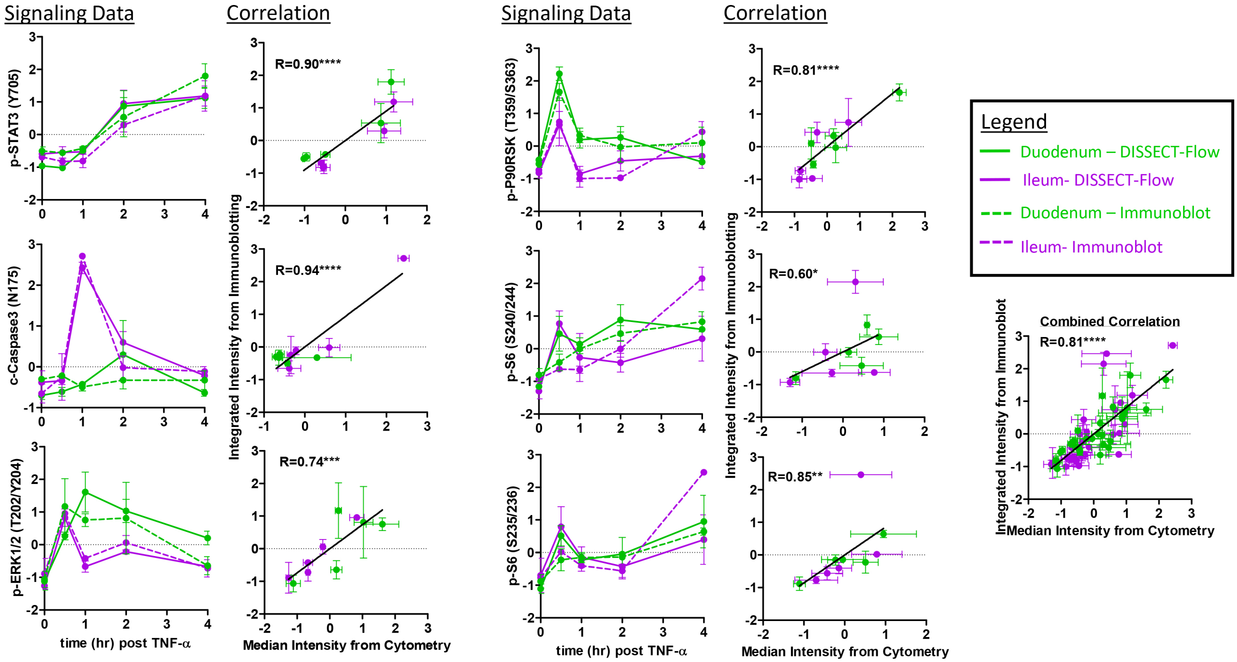

Appendix Figure S7: Quantitative comparison between single cell cytometric data and immunoblotting data of phospho-protein signaling markers. For description, see Figure 3. Error bars represent SEM from n=3 animals. * P ≤ 0.05, ** P ≤ 0.01, *** P ≤ 0.001, **** P ≤ 0.0001

Appendix Figure S8

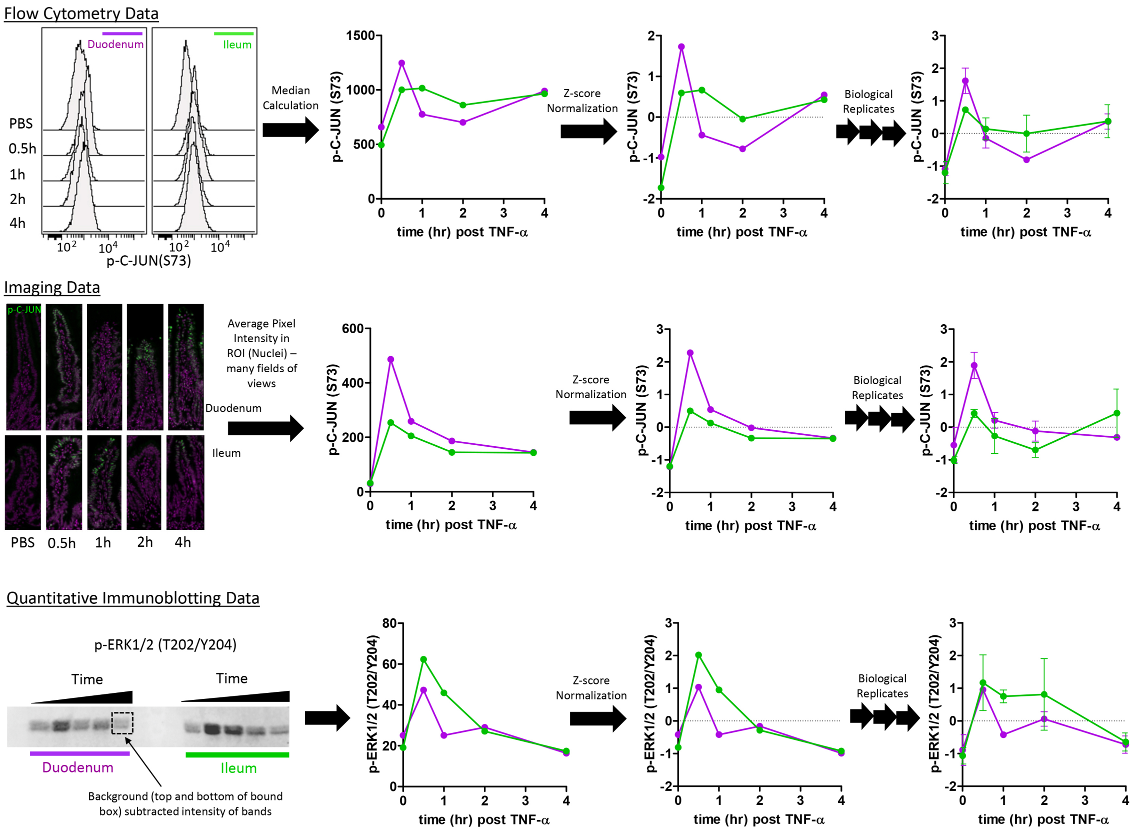

Appendix Figure S8: Methodology of quantitative comparison between experimental approaches. The median intensity of flow cytometry was used to represent whole tissue average. Quantitative data was mean centered and variance scaled (Z-score normalization) with 3 biological replicates for each data point. For IF imaging analysis, a nuclear region of interest (ROI) was constructed with Hoeschst staining or a cytoplasmic ROI was constructed using an area 5 pixels outside the nuclear mask. One of the two masks was used depending on whether the signal was nuclear or cytoplasmic. This segmentation procedure was used because whole cell segmentation cannot be accurately performed without multiple membrane markers in this tissue. For quantitative immunoblotting, integrated intensity was calculated while subtracting the background contained within the top and bottom borders of the bounding box.

Appendix Figure S9

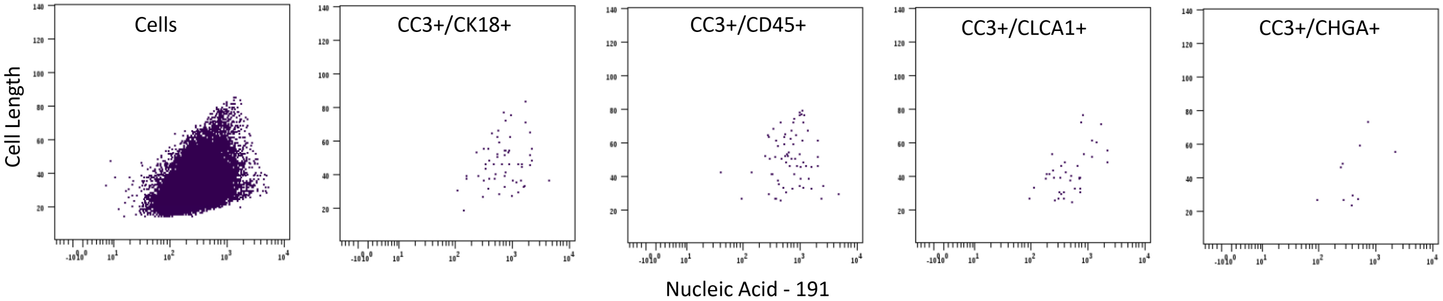

Appendix Figure S9: Verification of single cell acquisition using CyTOF. The first panel shows all cells designated as singlets analyzed, plotted as a function of nucleic acid staining and ion cloud size. The remaining panels show backgating of double-positive cells onto the same plot. An enrichment towards higher nucleic acid staining and larger ion cloud size, indicative of cell clusters, was not observed in the double-positive populations.

Appendix Figure S10

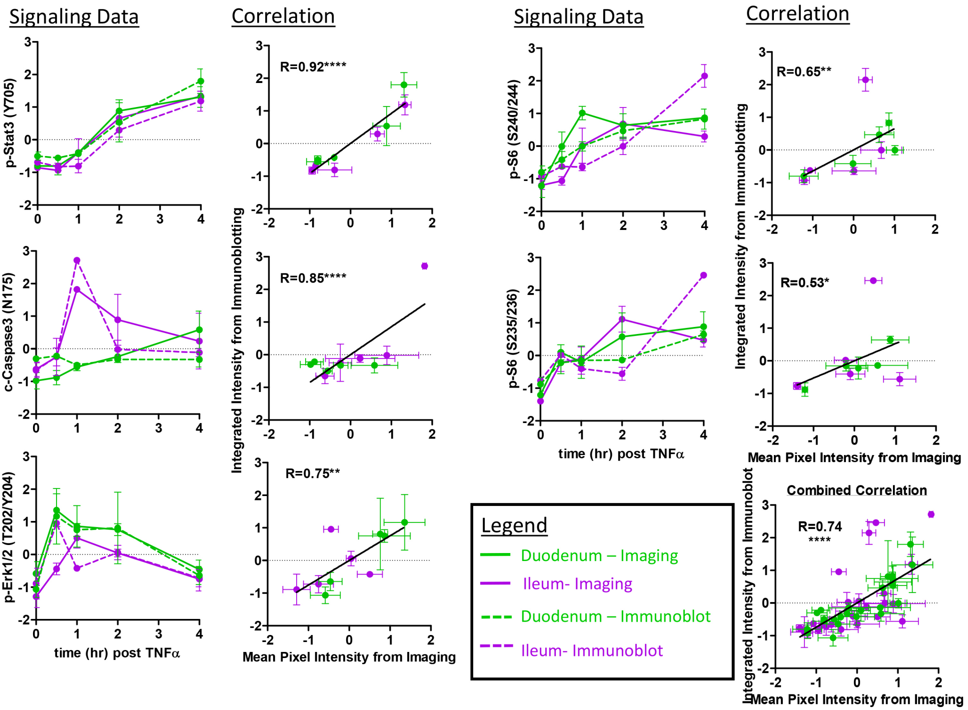

Appendix Figure S10: Quantitative comparison between imaging data and immunoblotting data of phospho-protein signaling markers. For description, see Figure 3. Error bars represent SEM from n=3 animals. * P ≤ 0.05, ** P ≤ 0.01, *** P ≤ 0.001, **** P ≤ 0.0001

Appendix Table S1

Epitope	Clone
p-C-JUN (Ser73)	D47G9
p-STAT3 (Tyr705)	D3A7
p-CREB (Ser133)	87G3
p-ERK1/2 (Thr202/Tyr204)	D13.14.4E
p-S6 (Ser240/244)	D68F8
p-S6 (Ser235/236)	N7-548
p-P38 (Thr180/Tyr182)	D3F9
p-P90RSK (Thr359/Ser363)	D1E9
p-MTOR (Ser2448)	D9C2
p-RB (Ser807/811)	J112-906
p-4E-BP1 (Thr37/46)	236B4
p-AKT (Ser473)	D9E
p-ATF2 (Thr71)	11G2
Cleaved Caspase 3 (Asp175)	polyclonal CS9661
DCLK1	EPR6085
p-EGFR (Tyr1068)	D7A5
Cytokeratin 20	D9Z1Z
CLCA1	EPR12554-88
Chromogranin A	Polyclonal SC1488
Cytokeratin 18	C-04
CD45	30-F11
Iκβα	L35A5
TNFR1	Polyclonal SC7859
TNFR2	D-2
LRIG1	Polyclonal AF3688
Na+/K+ ATPase	EP1845Y

Appendix Table S1: Antibody reagents used in this study.
