# Supplementary material for: Cytometry-based single-cell analysis of intact epithelial signaling reveals MAPK activation divergent from TNF-α-induced apoptosis in vivo
Source: Mol Syst Biol. 2015 Oct 30;11(10):835. doi: 10.15252/msb.20156282 (PMC4631206; doi:10.15252/msb.20156282)
Supplement: Supplementary file 2 — Expanded View Figures PDF [file msb0011-0835-sd2.pdf]

Expanded View Figures

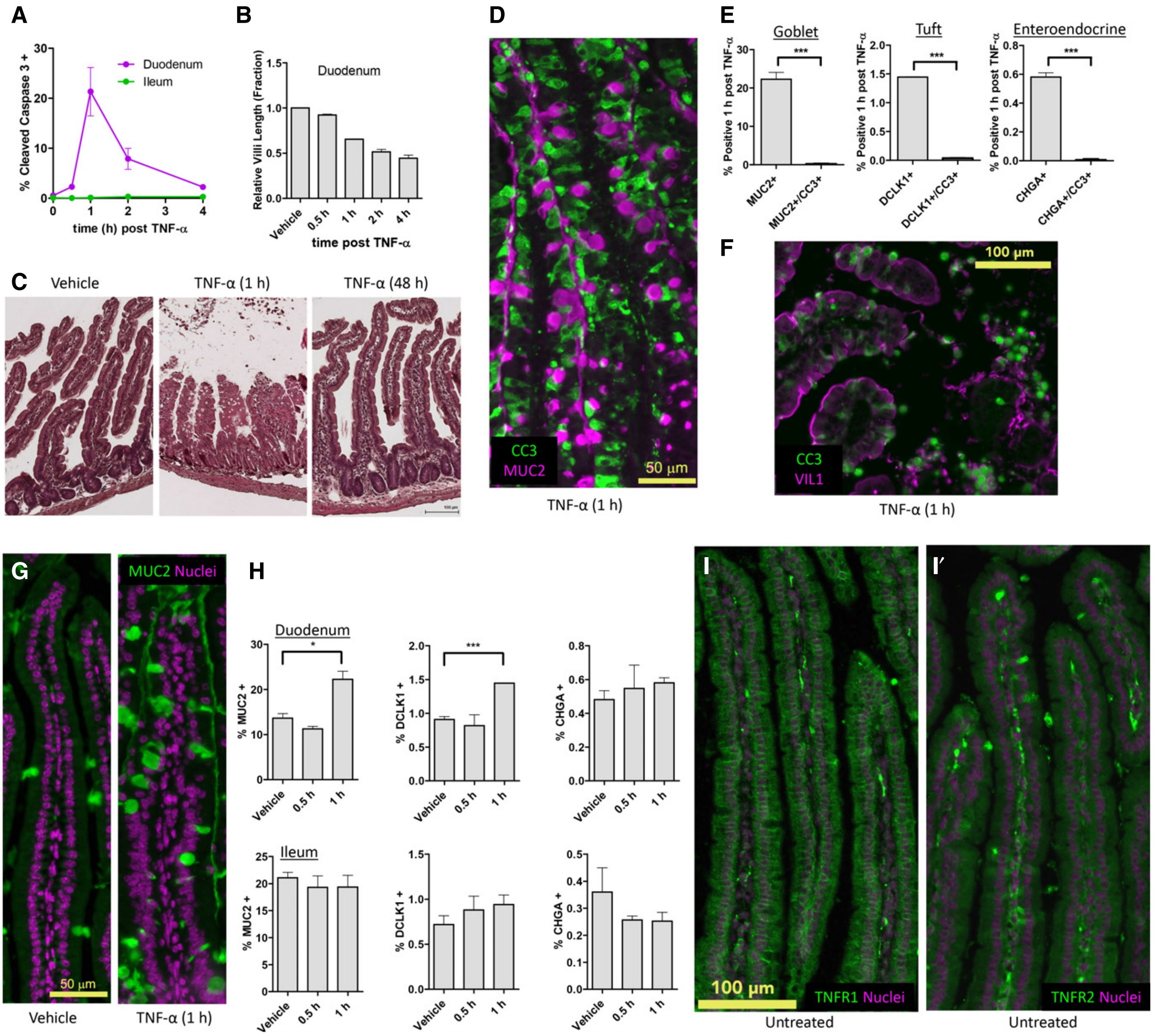

Figure EV1. TNF-α induces heterogeneous villus epithelial cell death.

A The fraction of CC3-positive cells identified by immunofluorescence (IF) image analysis over a time course of TNF-α induction. Error bars represent the standard error of the mean (SEM) from $n = 3$ mice per time point. Cell death percentage integrated over time is 34.25 ± 0.05%.

B Villi length quantified by image analysis, normalized to vehicle control. Error bars represent SEM from $n = 3$ animals.

C Morphology of villi 48 h post-TNF-α administration compared to vehicle control and 1 h post-TNF-α that exhibits severe villus blunting.

D Representative IF of non-overlapping MUC2 and CC3 1 h post-TNF-α administration.

E IF quantification of cells expressing villus epithelial cell markers only (MUC2—goblet cells, DCLK1—tuft cells, CHGA—enteroendocrine cells), or their co-expression with CC3 1 h post-TNF-α administration. Error bars represent SEM from $n = 3$ animals. Unpaired t-test was used to determine significance.

F IF of VIL co-localization with CC3 1 h post-TNF-α administration.

G Increased density of MUC2-positive cells detected by IF as cell death occurs.

H Quantification of mature villus cell types over time by IF image analysis using stereotypic markers (MUC2—goblet, DCLK1—tuft, CHGA—enteroendocrine), for both duodenum and ileum. Error bars represent SEM from $n = 3$ animals. Unpaired t-test was used to determine significance.

I IF for expression of (I) TNFR1 and (I′) TNFR2 in the villus.

Data information: $^{*}P \leq 0.05$, $^{***}P \leq 0.001$

© 2015 The Authors

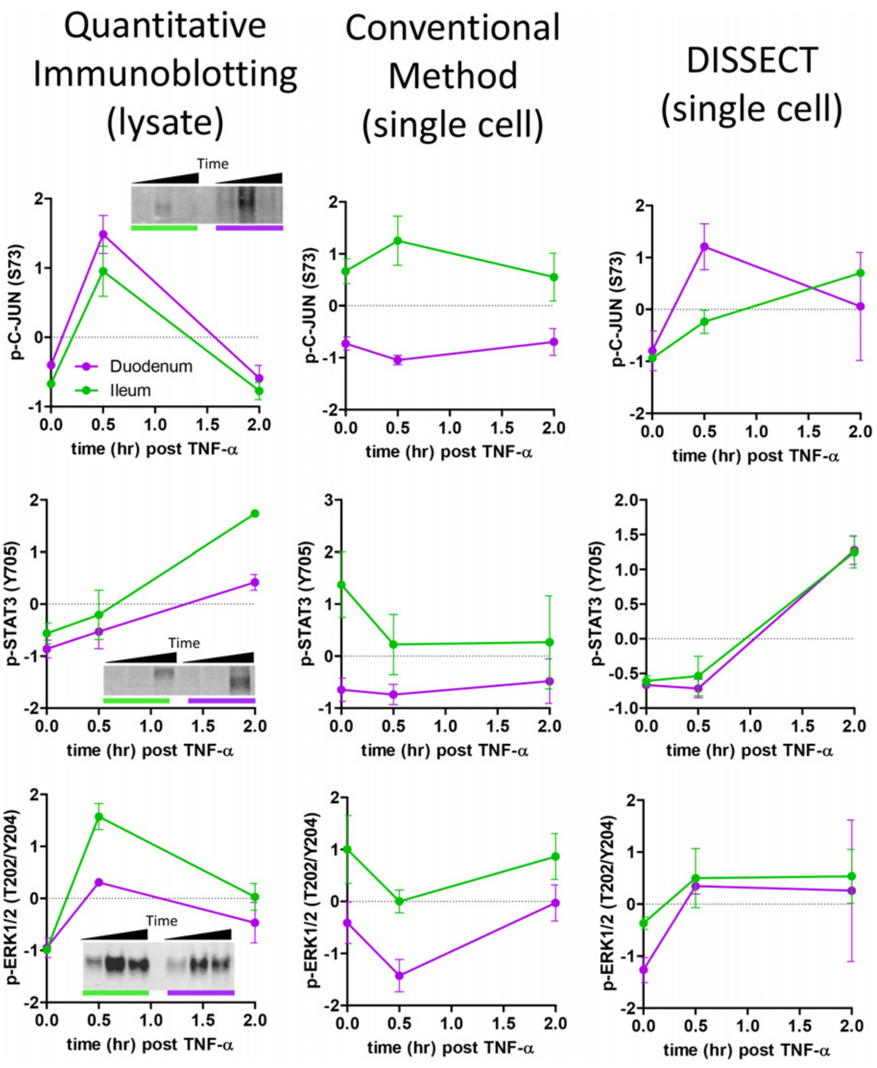

Figure EV2. DISSECT preserves native signal transduction during disaggregation.

Comparison between early (p-ERK, p-C-JUN) and late (p-STAT3) signaling data generated from quantitative immunoblotting, conventional disaggregation method followed by flow cytometry, and DISSECT followed by flow cytometry. Immunoblotting data represent integrated intensity of an immunoblot band. Flow cytometry data represents the median intensity of single-cell distributions. Data scales are Z-score values derived from mean centering and variance scaling of each time course experiment (Appendix Fig S8). Error bars represent SEM from biological duplicates.

 © 2015 The Authors

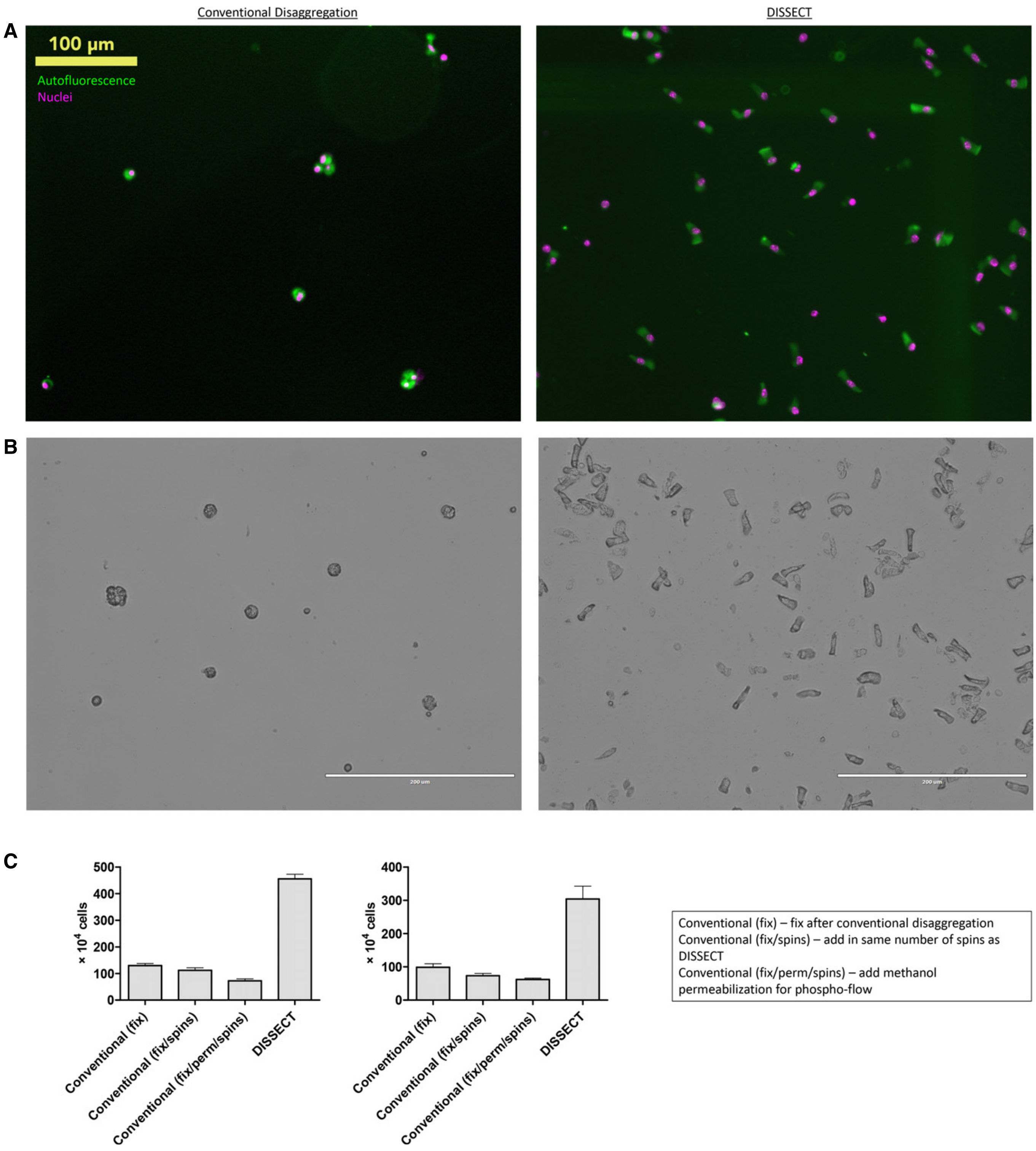

Figure EV3. DISSECT generates single-cell suspensions with comparable yield to the conventional disaggregation method.

A Whole field immunofluorescence image of single-cell suspensions post-disaggregation. The conventional sample was imaged prior to methanol permeabilization. Autofluorescence was used to image the cell body.

B Phase-contrast image of single-cell suspensions.

C Quantitative comparison of yields between different disaggregation conditions starting from the same amount of intestinal material from the same animal. Error bars represent SEM from *n* = 4 technical replicates. Two independent repeats were performed on different animals.

© 2015 The Authors

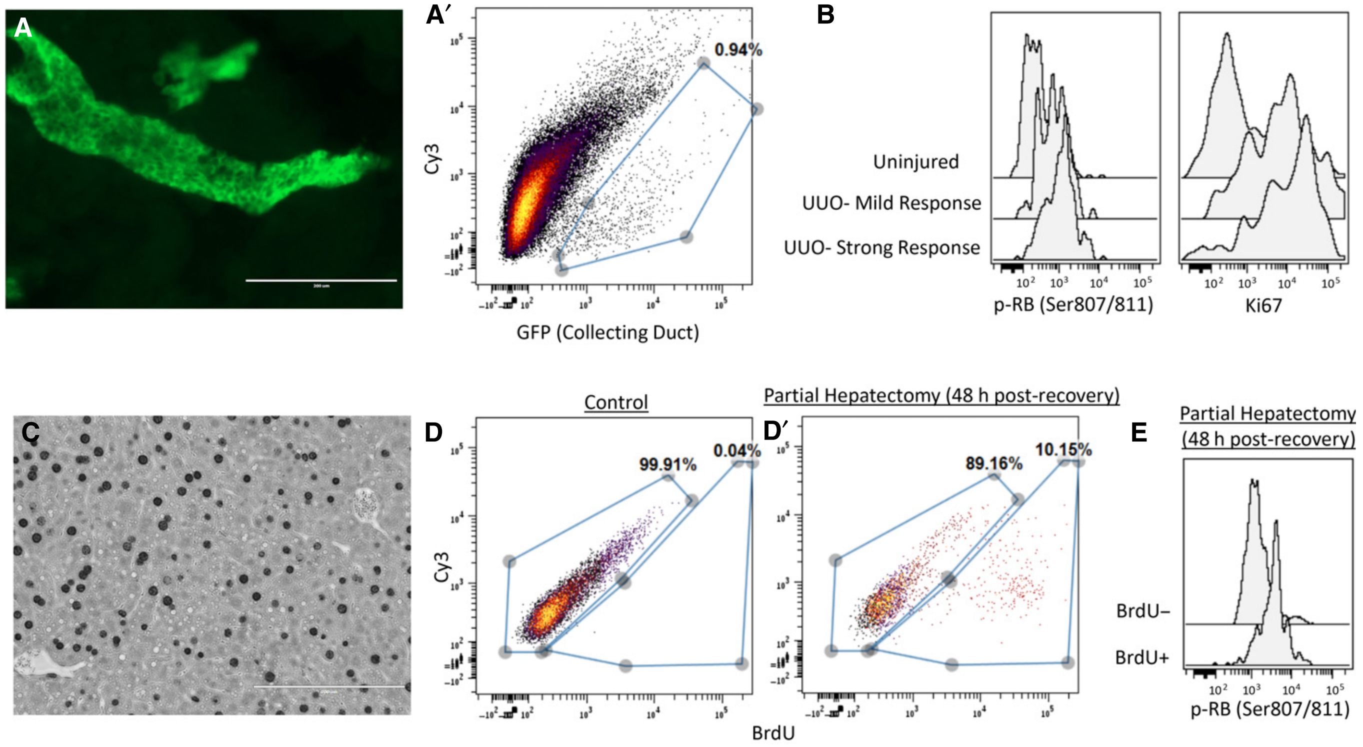

Figure EV4. DISSECT applied to other epithelial tissues also preserves intact signaling.

A Collecting duct epithelial cells of the kidney marked by GFP (Hoxb7-Cre; mT/mG). (A′) Single GFP^+ collecting duct cells in (A) detected by flow cytometry post-DISSECT.

B Proliferation of collecting duct cells 3 days post-unilateral ureteral obstruction (UUO) injury marked by Ki67, and proliferative p-RB signaling. Variation of proliferative response from different mice correlates with p-RB signaling.

C Hepatocyte proliferation post-partial hepatectomy labeled by BrdU.

D Single $BrdU^+$ cells detected by flow cytometry post-DISSECT in control (D) and post-partial hepatectomy (D′).

E p-RB proliferative signaling is enriched in $BrdU^+$-proliferating population compared to non-proliferating BrdU population.

 © 2015 The Authors

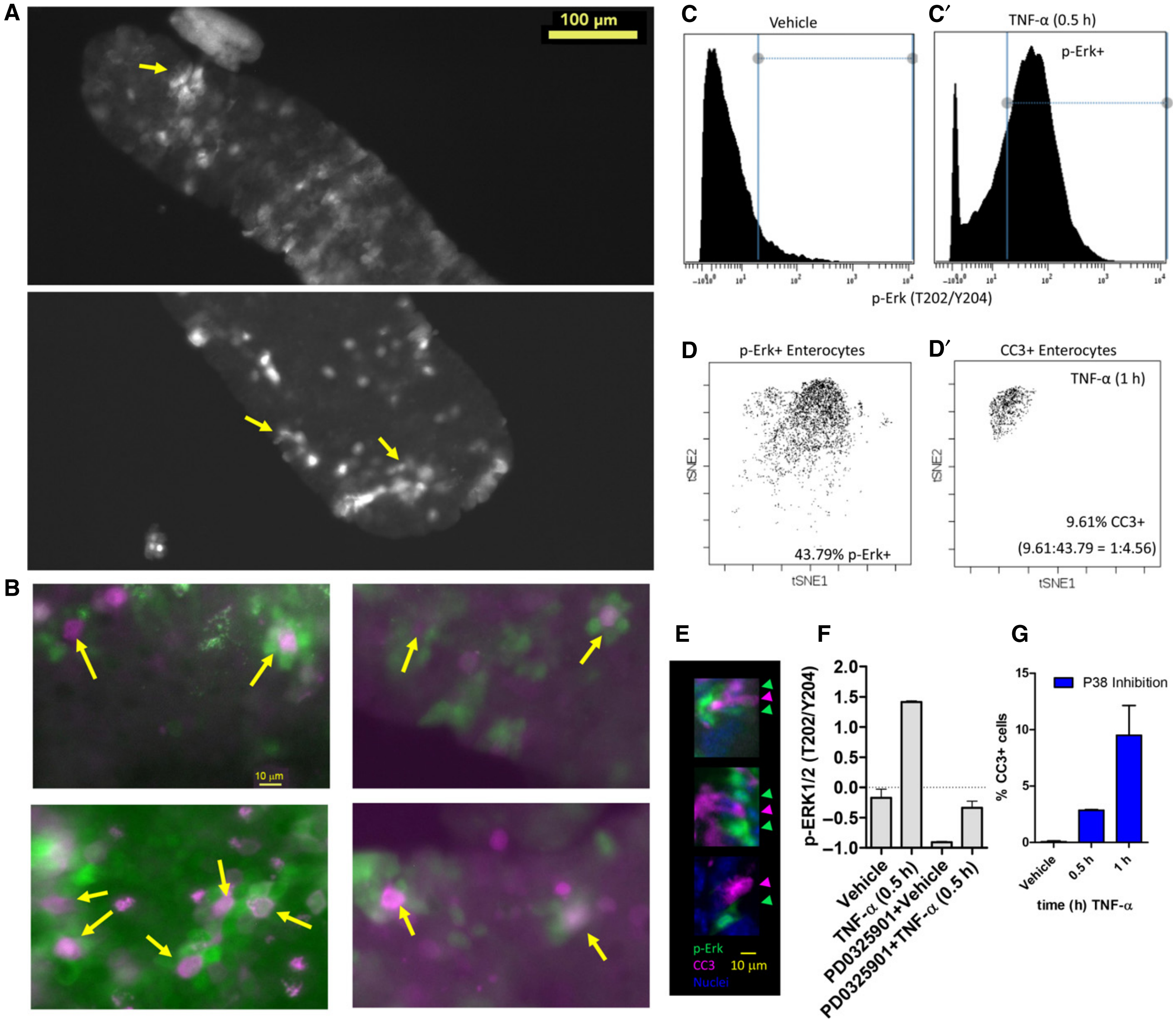

Figure EV5. Divergent p-ERK signaling cells are neighbors to dying cells.

A Distribution of p-ERK ring pattern along the whole villus, as indicated by the yellow arrows.

B More examples of $CC3^+$ cells surrounded by clusters of $p\text{-}ERK^+$ neighbors (yellow arrows).

C Gating of $p\text{-}ERK^+$ cells from CyTOF data. A vehicle control (C) and a TNF-α-treated sample (C′) were used to gate for cells with homeostatic p-ERK levels versus activated p-ERK levels, respectively.

D $p\text{-}ERK^+$ cells (D) and dying cells (D′) plotted in t-SNE space for cohort 1. The percentages of dying and $p\text{-}ERK^+$ cells were used to calculate ratio of dying to $p\text{-}ERK^+$ cells.

E Immunofluorescence of CC3 and p-ERK in duodenal tissue sections at 1 h post-TNF-α. $CC3^+$ cells (green arrowheads) were flanked by $p\text{-}ERK^+$ cells (magenta arrowheads).

F The efficacy of MEK inhibition assessed by p-ERK stimulation by TNF-α in the duodenum at 0.5 h. Error bars represent SEM of biological duplicates.

G Percentage of $CC3^+$ cells by flow cytometry in the context of P38 inhibition. Error bars represent SEM of biological duplicates.

© 2015 The Authors
